# Land use and mobility during the Neolithic in Wales explored using isotope analysis of tooth enamel

**DOI:** 10.1002/ajpa.23279

**Published:** 2017-07-28

**Authors:** Samantha Neil, Janet Montgomery, Jane Evans, Gordon T. Cook, Chris Scarre

**Affiliations:** ^1^ Department of Archaeology Durham University South Road Durham DH1 3LE United Kingdom; ^2^ NERC Isotope Geosciences Laboratory Keyworth Nottingham NG12 5GG United Kingdom; ^3^ Scottish Universities Environmental Research Centre Rankine Avenue East Kilbride G75 0QF United Kingdom

**Keywords:** development of farming, Neolithic, radiocarbon dates, strontium isotope analysis, Wales

## Abstract

**Objectives:**

The nature of land use and mobility during the transition to agriculture has often been debated. Here, we use isotope analysis of tooth enamel from human populations buried in two different Neolithic burial monuments, Penywyrlod and Ty Isaf, in south‐east Wales, to examine patterns of land use and to evaluate where individuals obtained their childhood diet.

**Materials and Methods:**

We employ strontium (^87^Sr/^86^Sr) and oxygen (δ^18^O) and carbon (δ^13^C) isotope analysis of enamel from adjacent molars. We compare strontium isotope values measured in enamel to locally bioavailable ^87^Sr/^86^Sr values. We combine discussion of these results with evaluation of new radiocarbon dates obtained from both sites.

**Results:**

The majority of enamel samples from Penywyrlod have strontium isotope ratios above 0.7140. In contrast, the majority of those from Ty Isaf have ^87^Sr/^86^Sr values below 0.7140. At Penywyrlod oxygen isotope ratios range between 25.9 and 28.2 ‰ (mean 26.7 ± 0.6 ‰, 1σ, *n* = 15) and enamel δ^13^C_carbonate_ values range between −18.0 and −15.0 ‰ (mean −16.0 ± 0.8 ‰, 1σ, *n* = 15). At Ty Isaf oxygen isotope ratios exhibited by Neolithic individuals range between 25.4 and 27.7 ‰ (mean 26.7 ± 0.6 ‰, 1σ, *n* = 15) and enamel δ^13^C_carbonate_ values range between −16.9 and −14.9 ‰ (mean −16.0 ± 0.6 ‰, 1σ, *n* = 15).

**Discussion:**

The strontium isotope results suggest that the majority of individuals buried at Penywyrlod did not source their childhood diet locally. One individual in this group has strontium isotope ratios that exceed all current known biosphere values within England and Wales. This individual is radiocarbon dated to the first few centuries of the 4^th^ millennium BC, consistent with the period in which agriculture was initiated in Wales: the results therefore provide evidence for migration during the transition to farming in Wales. In contrast, all individuals sampled from Ty Isaf post‐date the period in which agriculture is considered to have been initiated and could have sourced their childhood diet from the local region in which they were buried.

## 1 INTRODUCTION

The transition to farming in Britain during the 4^th^ millennium BC was associated with the introduction of non‐native domesticated species from the European mainland and the appearance of new cultural traditions and technologies, such as pottery manufacturing and burial monument construction. However, the mechanisms underlying this transition have been heavily debated. Both agriculture and traditions such as monument construction and pottery manufacturing were well established on the Continent at this time (e.g., Bradley, Haselgrove, Webley, & Vander Linden, 2015; Scarre, 2015; Tresset, 2015). Some authors suggest that local Mesolithic communities within Britain chose to adopt these practices, importing domesticated species from the near Continent during the early 4^th^ millennium BC (Cummings & Whittle, 2004, pp. 88–91; Cummings & Harris, 2011; Thomas, 2003, pp. 73, 2004, pp. 105, 2007, pp. 426, 2008, pp. 77, 2013, pp. 273). In contrast, others argue that these traditions were introduced by migration of farming communities to Britain from the European mainland (Anderson‐Whymark & Garrow, 2015; Collard, Edinborough, Shennan, & Thomas, 2010; Pailler & Sheridan, 2009, pp. 29; Rowley‐Conwy, 2011, pp. 443; Sheridan, 2004, 2007, pp. 465–467, 2010a; Whittle, Bayliss, & Healy, 2011, pp. 858–861). These authors suggest that a transformation in subsistence practices during the early 4^th^ millennium BC and a shift from hunting of wild animals and routine exploitation of marine foods toward heavy reliance on terrestrial domesticated resources, was a consequence of the arrival of farming groups (e.g., Richards & Hedges, 1999; Richards, Schulting, & Hedges, 2003; Richards & Schulting, 2006, pp. 453; Schulting, 2008, pp. 93–95; Serjeantson, 2014, pp. 261). The area of the European mainland from which such groups may have originated, however, remains the subject of debate (e.g., Anderson‐Whymark & Garrow, 2015; Sheridan, 2010a; Whittle et al., 2011, pp. 848–853). Radiocarbon dating suggests that Neolithic material culture and practices began to appear in different regions of Britain at different times (Bayliss, Healy, Whittle, & Cooney, 2011a, pp. 839). While agriculture is thought to have become established in south‐eastern England at approximately 4,000 BC, in other regions, such as Wales, Scotland and Ireland, Neolithic material culture and practices began to appear during the following two to three centuries (Bayliss et al., 2011a, 2011b; Cooney et al., 2011; Whittle et al., 2011, pp. 861–862). Recent comparative analysis of pottery could indicate that farming populations arrived from different regions of the European mainland at different times during the early 4^th^ millennium BC, as pottery found in eastern Britain appears to reflect influences from north‐eastern France and the Scheldt valley, while that found in Western Britain is considered to derive from traditions in Brittany and Lower Normandy, in north‐western France (Pioffet, 2015; cf. Sheridan, 2010b).

The nature of land use and residence patterns during the Neolithic in Britain has also been the subject of intense debate. Some authors suggest the first farmers in Britain were fully sedentary and argue that communities obtained the majority of their dietary resources, keeping livestock and cultivating crops, close to permanently occupied settlements (Bogaard et al., 2013, pp. 12589; Rowley‐Conwy, 2003, 2004, 2011). These authors place emphasis on the discovery of substantial timber buildings and argue that mobility of early farming communities was limited by the demands of cereal cultivation (Jones, 2000, 2005, pp. 172–173; Jones & Rowley‐Conwy, 2007; Rowley‐Conwy, 2000, 2003; Rowley‐Conwy & Legge, 2015).

Others, however, suggest that there was greater diversity in subsistence and settlement patterns. These authors highlight evidence for exploitation of a varied range of resources during the early Neolithic in Britain, including use of both wild plants and cereals (e.g., Bishop, Church, & Rowley‐Conwy, 2009, pp. 86, 90; Stevens, 2007, pp. 381–382) and suggest that evidence for cattle herding could indicate that some members of the community were residentially mobile (Thomas, 2013, pp. 411; e.g., Schulting, 2013, pp. 321; Serjeantson, 2011; Viner, 2011). A varied range of evidence for occupation may also be indicative of diversity in settlement systems during the early Neolithic in Britain (e.g., Anderson‐Whymark & Thomas, 2012; Brophy, 2015; Sheridan, 2013). In addition to the remains of substantial timber buildings, the presence of ephemeral structures, pits, lithic scatters and middens could indicate communities moved episodically between occupation sites that were located in different geographical areas to obtain their dietary resources (Garrow, Beadsmoore, & Knight, 2005, pp. 155; Whittle, 2003, pp. 43).

**Figure 1:**
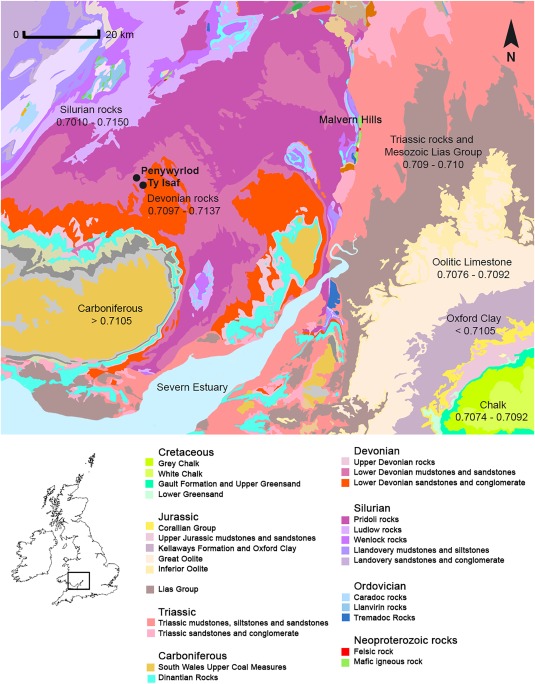
Map of bedrock geology illustrating sites and locations discussed in the text. Based on British Geological Survey and Ordnance Survey map data, reproduced with permission of the British Geological Survey and Ordnance Survey, © NERC/Crown copyright [2016]. Bioavailable ^87^Sr/^86^Sr ranges based on current measured values in Warham (2011: 70–96); Evans et al. (2010); Chenery et al. (2010); Montgomery et al. (2006) and Spiro et al. (2001). Locally bioavailable strontium isotope ratios on Devonian sandstones derived from measured values in plants collected within a radius of 10 miles, approximately 16 km, of Penywyrlod and Ty Isaf (Table 3)

In view of these debates we applied strontium (^87^Sr/^86^Sr) and oxygen (δ^18^O) isotope analysis of tooth enamel, which can be used to provide evidence for the location from which individuals may have obtained their childhood diet (e.g., Bentley, 2006; Montgomery, 2002, 2010; Slovak & Paytan, 2011, pp. 743–744), to explore patterns of land use and mobility during the Neolithic. Here we present the results of analysis of individuals from two burial monuments, the long cairns of Penywyrlod (Talgarth) and Ty Isaf, located less than 5 miles (8 km) apart amongst a cluster of monuments in the Black Mountains, Powys, south‐eastern Wales (Figure 1). Currently archaeological evidence for the nature of settlement in this region is very limited, few structural remains of Neolithic date have so far been discovered and surface lithic scatters dominate the archaeological record (Makepeace, 2006; Olding, 2000).

**Figure 2:**
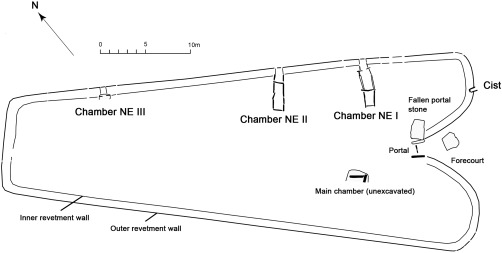
Penywyrlod (Talgarth) long cairn. Grey shaded areas are those which have been excavated. Image by Clwyd‐Powys Archaeological Trust; after Britnell and Savory 1984. Reproduced with permission of W.J. Britnell

**Figure 3:**
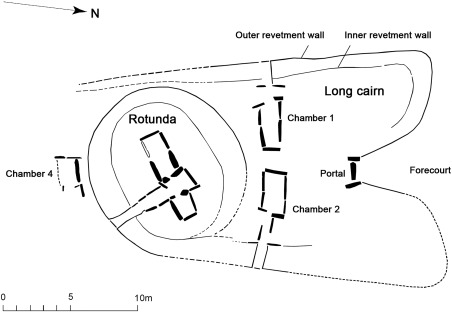
Ty Isaf long cairn. Image by Clwyd‐Powys Archaeological Trust; after Grimes 1939: 123. Reproduced with permission of W.J. Britnell

The long cairns of Penywyrlod (Talgarth) and Ty Isaf are part of the Cotswold‐Severn group, a concentration of burial monuments located in the regions around the Severn Estuary: south‐east Wales, the Cotswolds, Somerset and Wiltshire in southern Britain (Darvill, 2004, pp. 71–72). Although sharing a common geographical distribution, long cairns within the Cotswold‐Severn group are diverse in form. Penywyrlod (Talgarth) is a substantial long cairn over 60 metres in length. Partial excavation of the north‐eastern side of the monument revealed three lateral chambers which contained co‐mingled and disarticulated human remains (Figure 2, Luff, Brothwell, & O'Connor, 1984; Savory, 1973, pp. 187, 1984). Following excavation a sample of human bone recovered from one of the excavated chambers (chamber NEII) was radiocarbon dated to between 3960 and 3640 cal BC (95% confidence, OxCal v. 4.2; Britnell & Savory, 1984, pp. 29). In contrast, Ty Isaf is approximately 30 metres in length and is a composite monument consisting of two distinct elements: a long cairn with two opposing lateral chambers (chambers 1 and 2; Figure 3 over page) and a circular rotunda containing a transepted passage grave (chamber 3; Figure 3; Grimes, 1939, pp. 123–124). Human remains within the transepted passage grave in the rotunda date from the mid 4^th^ to earlier 3^rd^ millennium BC (Bayliss et al., 2011b, pp. 537, 546–547, see Radiocarbon Dating below).

### 1.1 Strontium isotope analysis: Principles

Use of strontium (^87^Sr/^86^Sr) isotope analysis for geographic provenancing is based on the principle that ^87^Sr/^86^Sr varies geographically depending on the age and composition of bedrock (Dicken, 2005, pp. 42–43; Faure, 1986, pp. 183–199; Faure & Mensing, 2005, pp. 76) and that humans derive strontium in their diet from biosphere sources (e.g., Bentley, 2006, pp. 141; Montgomery, 2002, pp. 17, 24, 36, 2010, pp. 328). Strontium weathers from rocks, entering soils and ground waters where it becomes available to plants and is transferred up the food chain (Capo, Stewart, & Chadwick, 1998, pp. 202–203), and incorporated into the mammalian skeleton. Tooth enamel is composed of carbonated hydroxyapatite, Ca_10_(PO_4_)_6_(OH)_2_ (Hillson, 2005, pp. 146–147); the calcium phosphate lattice, however, permits incorporation of other ions, such as strontium which is substituted for calcium (Johnson, Armstrong, & Singer, 1966; Rokita, Hermes, Nolting, & Ryczek, 1993). Strontium isotope analysis is undertaken on enamel as it is highly resistant to diagenesis (e.g., Budd, Montgomery, Barreiro, & Thomas, 2000; Trickett, Budd, Montgomery, & Evans, 2003). As conventionally measured ^87^Sr/^86^Sr values do not vary significantly between trophic levels (Graustein, 1989, pp. 492; e.g., Blum, Taliaferro, Weisse, & Holmes, 2000) and as enamel does not remodel once mineralized, strontium isotope ratios directly reflect sources to which an individual was exposed during tooth formation (Bentley, 2006; Montgomery, 2002). Strontium isotope ratios in modern plants and waters have been shown to vary with the age and composition of the underlying lithology (e.g., Evans, Montgomery, Wildman, & Boulton, 2010; Warham, 2011; Willmes et al., 2013). Comparison of ^87^Sr/^86^Sr values to mapped bioavailable values can therefore be used to evaluate whether an individual obtained dietary resources from the area in which they were later buried or whether they sourced their diet from a region further afield.

As indicated above there are particularly dense concentrations of Neolithic burial monuments in some regions of Britain, such as the Black Mountains in south‐eastern Wales. Isotope analysis of tooth enamel may assist in understanding why particular regions were chosen for monument construction: if individuals have values comparable to the local ^87^Sr/^86^Sr biosphere range it could suggest that monuments were located in areas used for settlement. Early Neolithic monuments contain the remains of multiple indivduals (see Section Materials and Methods, below). Comparison of the isotope ratios exhibited by different individuals within a monument may be used to infer whether they could have sourced their childhood diet from a similar geographical location. Likewise, comparison of isotope ratios in teeth that form at successive stages of childhood and adolescence can also be used to evaluate whether an individual obtained their diet from a similar geographical location throughout early life.

Both Penywyrlod and Ty Isaf are located on mudstones and sandstones formed during the early Devonian period (St Maughans Formation, BGS 2016). Samples of plants and water taken on Devonian lithology of similar age in Hereford, south‐east Gloucestershire, east Somerset and north Devon have previously recorded ^87^Sr/^86^Sr values between 0.7113 and 0.7129 (mean 0.7121 ± 0.0005, 1σ, *n* = 6, Chenery, Mueldner, Evans, Eckardt, & Lewis, 2010, pp. 155; Evans et al., 2010; Montgomery, Evans, & Wildman, 2006, pp. 1628). All currently available measured biosphere data therefore suggests that ^87^Sr/^86^Sr values above 0.7140 are not bioavailable on Devonian lithology (ibid). To provide further direct evidence for the range of locally bioavailable ^87^Sr/^86^Sr values at Penywyrlod and Ty Isaf the present study presents the results of analysis of modern plants growing on lithology of Devonian age within radius of 10 miles (approximately 16 km) of both sites (Section 2.2 Materials and Methods).

Samples of plants and water taken on lithology of Silurian age in Wales have previously recorded values between 0.7010 and 0.7150 (mean 0.7120 ± 0.0012, 1σ, *n* = 16) while those taken on Ordovician rocks have recorded a range of values between 0.7093 and 0.7152 (mean 0.7128 ± 0.0002, 1σ, *n* = 64, Evans et al., 2010; Montgomery et al., 2006, pp. 1628; Shand, Darbyshire, Gooddy, & Haria, 2007, pp. 254, 256), highlighting the potential for heterogeneity in bioavailable strontium isotope ratios on lithology of Lower Palaeozoic age. Samples of deep groundwater taken on lithology of Lower Palaeozoic age in central Wales can give values up to 0.7152 (Shand et al., 2007, pp. 256), however, all presently available biosphere data suggests that lithologies of this age in Wales are routinely associated with lower mean values.

The oldest rocks in England and Wales crop out approximately 16 miles (26 km) to the north‐east of Penywyrlod and Ty Isaf at Stanner Hill close to the English‐Welsh border and just over 35 miles (56 km) to the east within the Malvern Hills in the counties of Herefordshire and Worcestershire (BGS, 2016; Woodcock & Strachan, 2012, pp. 140). A single sample of plants growing on rocks of Neoproterozoic age in the Malvern Hills produced the highest bioavailable ^87^Sr/^86^Sr value currently known within England and Wales (0.7162, Chenery et al., 2010, pp. 155). However, as rocks of this age crop out in a very limited geographical area, values such as this have so far remained difficult to reproduce (Lucie Johnson, pers. comm.), with water sampled close to the Malvern Hills having given a value of 0.7132 (Montgomery et al., 2006, pp. 1628) and plants recording a mean ^87^Sr/^86^Sr value of 0.7128 ± 0.0040 (2σ, *n* = 13, Chenery et al., 2010, pp. 155–156).

### 1.2 Oxygen and carbon isotope analysis: Principles

The oxygen isotope composition of water also varies geographically with factors such as temperature, latitude, altitude and distance from the coast (e.g., Gat, 2010; Mook, 2005, pp. 89–98). Britain receives most of its rainfall from a westerly direction and contemporary groundwaters in western Britain therefore record higher δ^18^O values than those in eastern Britain (Darling, Bath, & Talbot, 2003, pp. 189–190). The use of oxygen isotope analysis for geographic provenancing of human individuals is based on the premise that, although there is some contribution from respiratory oxygen and chemically‐bound oxygen in food, a significant component of the δ^18^O values in mammalian bioapatite derives from ingested fluids which can therefore reflect values in drinking water (e.g., Daux et al., 2008, pp. 1146; Levinson, Luz, & Kolodny, 1987; Longinelli, 1984; Luz, Kolodny, & Horowitz, 1984; Luz & Kolodny, 1985; Kirsanow & Tuross, 2011; Podlesak et al., 2008). Evans, Chenery, and Montgomery (2012, pp. 759) argue that a statistically significant difference in the mean δ^18^O values of tooth enamel from multi‐period archaeological populations buried in western Britain (18.2 ‰ ± 1.0 ‰, 2σ) to those buried within eastern Britain (17.2 ± 1.3 ‰, 2σ) reflects differences in the oxygen isotope composition of local drinking water between these two regions. A recent overview of all currently published human tooth enamel and bone bioapatite data demonstrates that oxygen isotope values exhibited by human archaeological populations are correlated with environmental factors such as latitude, longitude, altitude (Lightfoot & O'Connell, 2016). However, it also highlights the way in which the oxygen isotope ranges of populations buried in different areas of temperate Europe can overlap (ibid.). As such, δ^18^O values exhibited by individuals excavated on the immediate European mainland may be similar to those recorded in Britain (e.g., see Brettell, Evans, Marzinzik, Lamb, & Montgomery, 2012a, pp. 127).

The above δ^18^O ranges for multi‐period archaeological populations buried in Britain were determined from the phosphate ( ${\text{PO}}_{4}^{3–}$) fraction of tooth enamel. However, just as calcium can be replaced by strontium in the bioapatite lattice (see above), both the hydroxyl (OH)^‐^ and phosphate ( ${\text{PO}}_{4}^{3–}$) groups can also be substituted by carbonate ( ${\text{CO}}_{3}^{2–}$) (LeGeros, 1991, pp. 119–121; Sønju Clasen & Ruyter, 1997). The structural carbonate ( ${\text{CO}}_{3}^{2–}$) fraction of tooth enamel of Holocene age is considered to be resistant to diagenesis (e.g., Koch, Tuross, & Fogel, 1997; Zazzo, 2014) and was analysed in the present study. δ^18^O values of the δ^18^O_phosphate_ and δ^18^O_carbonate_ fractions are considered to be well correlated and conversion between the two was therefore undertaken using the equation of Chenery, Pashley, Lamb, Sloane, and Evans (2012; see Section 2.2 Materials and Methods, below). The interpretation of results must, however, give consideration to the potential influence of culinary practice (e.g., stewing foods and brewing: Brettell, Montgomery, & Evans, 2012b; Daux et al., 2008, pp. 1144), or the consumption of fluids that have undergone fractionation through biological processes (e.g., cow's milk, Camin, Perini, Colombari, Bontempo, & Versini, 2008, pp. 1695; Kornexl, Werner, Rossmann, & Schmidt, 1997, pp. 22; Lin, Rau, Chen, Chou, & Fu, 2003, pp. 2193), on the δ^18^O values of ingested fluids (Lightfoot & O'Connell, 2016). Breast milk can have a higher δ^18^O value relative to fluids consumed by the mother and teeth which form while an infant is being breast fed (e.g., deciduous molars) may therefore have higher values than teeth that form later in childhood (Britton, Fuller, Tütken, Mays, & Richards, 2015, pp. 8; Roberts, Coward, & Ewing, 1988, pp. 625; Wright & Schwarcz, 1998, pp. 14).

Isotope analysis of the structural carbonate fraction of enamel simultaneously yields carbon isotope ratios (δ^13^C_carbonate_) which provide additional dietary information. Use of carbon isotope analysis for this purpose exploits the large natural variation in δ^13^C values of plants that use the two dominant (C_3_ or C_4_) photosynthetic pathways during fixation of CO_2_ energy, and contrasting δ^13^C values of terrestrial C_3_ and marine ecosystems (Schwarcz & Schoeninger, 2011; Sponheimer & Cerling, 2014). Current understanding of dietary composition in the European Neolithic is primarily based on analysis of δ^13^C and δ^15^N values in bone collagen, which predominantly reflect the protein component of the diet and support routine exploitation of C_3_ terrestrial sources of protein during the early Neolithic in Britain (e.g., Richards & Hedges, 1999, pp. 893; Richards et al., 2003). In contrast, δ^13^C_carbonate_ values in bioapatite reflect the isotope composition of the diet as a whole, including lipids and carbohydrates (Ambrose & Norr, 1993, pp. 2; Jim, Ambrose, & Evershed, 2004). Individuals who obtain the majority of their diet from C_3_ terrestrial sources may be predicted to have δ^13^C_carbonate_ values between approximately −17.0 to −14.0 ‰ (Froehle, Kellner, & Schoeninger, 2012; Kellner & Schoeninger, 2007).

### 1.3 Radiocarbon dating

The program of radiocarbon dating conducted by Bayliss et al. (2011b, pp. 537, 546–547) found that the majority of sampled individuals buried in the transepted passage grave within the rotunda at Ty Isaf dated from the mid to late 4^th^ millennium BC. However, individuals buried in the adjoining long cairn (Figure 3) have not been radiocarbon dated. The excavator was unable to determine which of the two monuments was constructed first: it is possible that both of these monuments were in use at the same time; alternatively, the rotunda could have been inserted into the long cairn at a later date (Grimes, 1939, pp. 137–138). In addition, radiocarbon dating of bone from the rotunda also revealed the presence of several individuals dated to the earlier third millennium BC (OxA‐14248, 2900–2670 cal BC and OxA‐14250, 2860–2490 cal BC, Bayliss et al., 2011b, pp. 537; OxCal v4.2, IntCal13), which is argued to indicate that Ty Isaf was the focus of secondary burial activity during the later Neolithic (ibid.). Fragments of Bronze Age pottery found during excavation could also suggest the site was a focus for activity during later periods (Grimes, 1939, pp. 125, 130 and 135–136). All burials sampled by the present study from Ty Isaf were therefore radiocarbon dated to assist in evaluating whether individuals buried in the long cairn are contemporary with those in the rotunda and whether the site remained of importance to communities beyond the 4^th^ millennium BC.

## 2 MATERIALS AND METHODS

### 2.1 Sample selection

Burial assemblages from Penywyrlod and Ty Isaf long cairns consist of highly fragmentary disarticulated and co‐mingled human remains. Care therefore had to be taken to avoid the potential for duplication of isotope results through inadvertent sampling of cross matching fragments of dentition (e.g., mandibular and maxillary) that could belong to the same individual. Only teeth from the left mandibular dentition were selected for sampling. Nine different human individuals from Ty Isaf were sampled (Table 1). Left mandibular dentition from nine different individuals from Penywyrlod was sampled (Table 2). In addition, two pre‐existing chips of core enamel taken in 2003–2004 from right mandibular third molars from Penywyrlod by a project unrelated to the present study were also analysed to obtain isotope ratios: 74.23H/9.5.19/P27 and 74.23H/9.16/P27 (Table 2).

**Table 1:** Results of radiocarbon dating and ^87^Sr/^86^Sr, δ^18^O_carbonate_ and δ^13^C_carbonate_ analysis of individuals excavated from Ty Isaf

Accession number	Location/context	Age category/age at death	Tooth	Laboratory number (SUERC)	δ^13^C ‰ VPDB collagen	Radiocarbon age (BP)	Calibrated date range (cal BC, 95% confidence)	Sr ppm (mg/kg)	^87^Sr/^86^Sr	δ^13^C_carbonate_ ‰ VPDB enamel	δ^18^O_carbonate_ ‰ VPDB	δ^18^O_carbonate_ ‰ VSMOW	δ^18^O_phosphate_ ‰ VSMOW
39.190/317	Undocumented	Adult	LM2	SUERC‐57795 (GU36260)	−20.7	4772 ± 30	3650–3380	50	0.71181	−15.7	−3.4	27.4	18.6
			LM3					35	0.71284	−16.2	−3.6	27.2	18.4
39–190/312	Undocumented	Adult	LM2					71	0.71300	−16.1	−4.6	26.2	17.4
			LM3	SUERC‐57796 (GU36261)	−20.8	4755 ± 31	3640–3380	83	0.71265	−15.6	−4.3	26.5	17.7
39.190/148b	Chamber 1, East compartment	Adult	LM2	SUERC‐57787 (GU36255)	−21.0	4680 ± 30	3630–3370	80	0.71238	−16.3	−4.1	26.7	17.9
			LM3					102	0.71372	−16.2	−4.2	26.6	17.8
39.190/310	Chamber 2, South side of entrance passage	Adult	LM2	SUERC‐57786 (GU36254)	−20.7	4672 ± 31	3630–3360	68	0.71436	−15.4	−4.1	26.7	17.9
			LM3					76	0.71330	−15.1	−4.6	26.2	17.4
39.190/201	Undocumented	Adult	LM2	SUERC‐57794 (GU36259)	−20.6	4672 ± 31	3630–3360	78	0.71328	−15.3	−4.3	26.5	17.6
			LM3					97	0.71282	−16.0	−4.4	26.4	17.5
39.190/58	Chamber 1, North East corner of East compartment	Sub‐adult (> 12 years)	LM2	SUERC‐57784 (GU36252)	−20.9	4616 ± 30	3520–3340	79	0.71524	−14.9	−5.4	25.4	16.5
39.190/148a	Chamber 1, East compartment	Adult	LM2	SUERC‐57785 (GU36253)	−21.2	4594 ± 30	3500–3120	222	0.71059	−16.7	−3.1	27.7	19.0
			LM3					76	0.71351	−16.9	−4.0	26.7	17.9
39.190/59	Undocumented	Adult	LM2	SUERC‐57790 (GU36258)	−20.6	4430 ± 33	3330–2920	52	0.71177	−16.8	−3.4	27.4	18.6
			LM3					61	0.71017	−16.2	−3.9	26.9	18.1
39.190/324	Chamber 2, Outside chamber on North side	Child (3–4 years)	LDM1					59	0.71409	−15.2	−3.6	27.2	18.4
			LDM2	SUERC‐57789 (GU36257)	−20.7	3309 ± 32	1670–1500	64	0.71458	−15.6	−3.6	27.2	18.4
39.190/cow	Chamber 1, West compartment	Unknown	PM3 Cervix (0.5 ‐ 5.0 mm)	SUERC‐57788 (GU36256)	−22.9	4039 ± 30	2840–2470	126	0.71312				
			PM3 Cusp (12.5 −17.5 mm)					155	0.71304				

Information on the contexts of sampled specimens (where available) is as recorded on labelling and documentation associated with the collections in the National Museum Wales. All human teeth derive from mandibular dentition: L = left; DM1 = deciduous first molar; DM2 = deciduous second molar; M2 = permanent second molar; M3 = permanent third molar. PM3 = loose third permanent premolar of a cow.

**Table 2:** Results of radiocarbon dating and ^87^Sr/^86^Sr, δ^18^O_carbonate_ and δ^13^C_carbonate_ analysis of individuals from Penywyrlod (Talgarth)

Museum accessionnumber	Location/context	Age category/age at death	Tooth	Laboratory number (SUERC)	δ^13^C ‰ VPDB collagen	Radiocarbon age (BP)	Calibrated date range (cal BC, 95% confidence)	Sr ppm (mg/kg)	^87^Sr/^86^Sr	δ^13^C_carbonate_ ‰ VPDB enamel	δ^18^O_carbonate_ ‰ VPDB	δ^18^O_carbonate_ ‰ VSMOW	δ^18^O_phosphate_ ‰ VSMOW
74.23H/9.18/P20	Cist in forecourt	Child (4 years)	LDM2					53	0.71583	−16.0	−2.7	28.2	19.4
74.23H/9.23/P21	NE chamber II	Adult	LM2					58	0.71459	−15.1	−3.6	27.2	18.4
			LM3					41	0.71480	−15.0	−3.7	27.1	18.2
74.23H/9.23/P22	NE chamber II	Adult	LM2					64	0.71501	−15.9	−4.4	26.4	17.5
			LM3					43	0.71390	−16.4	−4.3	26.5	17.7
74.23H/9.5.11/P23	NE chamber II, half way along chamber on south side	Sub‐adult (> 12 years)	LM2					52	0.71406	−15.8	−4.8	26.0	17.1
74.23H/9.2.3/P23	NE chamber II, north‐east end of north‐west side	Adult	LM2	SUERC‐63414 (GU38967)	−20.6	4888 ± 37	3770–3630	78	0.71702	−15.3	−4.3	26.5	17.7
			LM3					62	0.71653	−15.8	−4.9	25.9	17.0
74.23H/9.5.1/P24	NE chamber II	Adult	LM2					89	0.71473	−16.0	−4.0	26.8	17.9
			LM3					42	0.71335	−16.5	−4.2	26.6	17.8
74.23H/9.7/P25	NE chamber III	Child (2 years)	LDM2					97	0.71323	−18.0	−3.3	27.5	18.7
74.23H/9.7/P26	NE chamber III	Sub‐adult (> 12 years)	LM2					41	0.71441	−15.2	−4.1	26.7	17.9
74.23H/9.7/P27	NE chamber III	Sub‐adult (> 12 years)	LM3					86	0.71508	−17.1	−3.6	27.2	18.4
74.23H/9.5.19/P27	NE chamber II	Adult	RM3					34	0.71417	−16.1	−4.6	26.2	17.4
74.23H/9.16/P27	NE chamber II, west end of chamber, south side of paving slab	Adult	RM3					65	0.71472	−16.2	−4.8	26.0	17.1

All teeth sampled derive from mandibular dentition: L = left; R= right; DM2 = deciduous second molar; M2 = permanent second molar; M3 = permanent third molar. Location and context of specimens is as stated on documentation associated with the collection held by the National Museum Wales.

Information on the contexts of sampled specimens (where available) is given on the labelling and documentation associated with the collections now stored in the National Museum Wales and is provided in Tables 1 and 2. No dentition attributed to the rotunda monument at Ty Isaf could be located that met the criteria for sampling specified above. Four of the specimens from Ty Isaf lacked documentation detailing their excavation context (those listed in Table 1 as being of 'undocumented' context). These specimens could either have been excavated from the rotunda or the lateral chambers of the long cairn at Ty Isaf.

The human burial assemblage from Penywyrlod was recovered during a partial rescue excavation that was instigated following damage to the monument (Savory, 1973, 1984). It therefore represents a sample of what may be present at the site. The current study undertook analysis of tooth enamel from individuals buried in two of the three excavated chambers (Chambers NE II and NE III) and in a cist in the forecourt, within the lower revetment wall of the south‐east horn of the monument (Figure 2). Few remains were recovered during excavation of Chamber NE I (Savory, 1984, pp. 18; Britnell & Savory, 1984, pp. 6) and no dentition from this chamber was available that met the criteria for sampling specified above. During assessment of the collections it was found that several specimens from Penywyrlod possessed the same generic museum accession number. To differentiate between specimens, each was assigned an additional unique code, corresponding to the page number on which the specimen is listed in the sampling application made to the National Museum Wales (e.g., P21 refers to page 21 of the sampling application).

The sex of the majority of sampled individuals cannot be determined with confidence owing to the fragmentary nature of the assemblages and disarticulation of cranial remains from other skeletal elements used for sex attribution. Facial reconstruction commissioned by the National Museum Wales for the purpose of gallery display suggests individual 74.23H/9.23/P22 is male. Wherever possible, the approximate age of individuals at death (Tables 1 and 2) was determined from stage of dental eruption and tooth root development, following AlQahtani, Hector, and Liversidge (2010). Individuals who have fully erupted permanent dentition with fully formed third molar tooth roots are denoted as 'adult' in Tables 1 and 2. Where possible, consecutively mineralizing molars were sampled to compare isotope ratios at different stages of childhood: formation of the second molar crown occurs between approximately 2.5 ± 0.5 years and 8.5 ± 0.5 years of age (AlQahtani et al., 2010; Hillson, 2014, pp. 31, 55–56). Timing of third molar formation is more variable (Liversidge, 2008, pp. 313), with initial cusp formation from approximately 8.5 ± 0.5 years of age and crown completion by approximately 14.5 ± 0.5 years (AlQahtani et al., 2010). Strontium and oxygen isotope analysis was undertaken on samples of bulk enamel, and isotope ratios therefore represent the weighted average of all dietary sources exploited during the period in which the enamel was forming (Montgomery, 2010, pp. 333). An enamel chip of approximately 20–30mg in weight from each tooth was utilized for strontium isotope analysis and of approximately 10mg in weight for oxygen isotope analysis.

A limited number of fragmentary animal remains were also recovered during the excavation of Ty Isaf (Cowley, 1939, pp. 141–142), including a loose cattle third permanent premolar tooth (Table 1). This was sampled to compare strontium isotope ratios with those of the human burial group. Although formation of the tooth crown proceeds from cusp to cervix (Hillson 2005, pp. 156), complexity in the process of enamel maturation and averaging of strontium in the body pool prior to incorporation into enamel may limit the study of ^87^Sr/^86^Sr values at high chronological resolution in human populations (e.g., Montgomery, Evans, & Horstwood, 2010). In bovine high crowned (hypsodont) teeth it may, however, be possible to detect variation in strontium isotope ratios by taking spatially separated samples along the axis of tooth formation (e.g., Viner, Evans, Albarella, & Parker Pearson, 2010). One sample of enamel, of a similar size to those sampled from each human tooth, was therefore excised from the top (cusp) and one at the bottom (cervix) of the tooth crown. As the earliest forming enamel at the cusp can be eliminated through dental attrition the location from which these samples were excised was recorded in millimetres with respect to the cervical margin (Table 1). The sampled cattle tooth was a loose permanent third molar. In modern cattle the third permanent premolar crown begins to form at approximately 11–12 months of age and formation of the crown of this tooth is complete by approximately 24–30 months (Brown, Christofferson, Massler, & Weiss, 1960).

### 2.2 Sample preparation and laboratory analysis

Initial preparation of enamel samples for isotope analysis was undertaken in the laboratories at Durham University following procedures developed by Montgomery (2002, pp. 131–138). The enamel surface was mechanically cleaned using tungsten carbide dental burrs and a flexible diamond edged rotary saw was then used to excise a chip of core enamel. To remove any adhering dentine, exposed surfaces of the enamel chip were again thoroughly abraded using dental burrs. Resulting chips of core enamel were transferred to clean sealed containers. Dental saws and burrs were cleaned ultrasonically for five minutes and rinsed three times in high purity de‐ionized water between preparation of samples.

^87^Sr/^86^Sr analysis was undertaken in the Class 100, HEPA‐filtered laboratory facilities at the Natural Environment Research Council Isotope Geosciences Laboratory (Keyworth, Nottingham, England). Dried plant samples were lightly crushed and placed into pre‐cleaned pressure vessels in a clean laboratory environment. They were digested using 8M HNO_3_ and a trace of hydrogen peroxide in a microwave oven at 175 °C for 20 minutes according to methods described in Warham (2011). Enamel chips were cleaned ultrasonically, rinsed in high purity (Millipore Alpha Q) water and dried. They were then weighed into pre‐cleaned Teflon beakers and spiked with a known amount of ^84^Sr tracer solution to obtain strontium concentrations. Each enamel chip was then dissolved in Teflon distilled 8M HNO_3_, then converted to chloride using 6M HCl, taken up in titrated 2.5M HCl and pipetted onto ion exchange chromatography columns. Strontium was separated with Dowex® (AG50‐X8) resin (200–400 mesh). Procedural blanks were below 150 pg. Samples were loaded on to Re filaments following the method adapted from Birck (1986, pp. 79). Strontium isotope composition and concentrations were determined by thermal ionisation mass spectrometry (TIMS) using a *ThermoTriton* automated multi‐collector mass spectrometer. To correct for fractionation during the process of mass spectrometry ^87^Sr/^86^Sr values are normalised to the accepted value for ^88^Sr/^86^Sr = 0.1194. During the period of this study the machine gave a ^87^Sr/^86^Sr value for the international standard, NBS 987, of 0.710253 ± 0.000012 (2σ, *n* = 350). An estimate of the reproducibility of strontium concentration (Sr ppm) is provided by replicate analyses of an aliquot of bone standard solution (NIST1486) which gave 7.22 ± 0.27 ppm (± 3.75%, 1σ, *n* = 16).

Initial preparation of core enamel chips for δ^18^O and δ^13^C analysis was undertaken at Durham University using the same methods employed above for strontium isotope analysis. Core enamel chips were then transferred to the laboratory facilities at the Natural Environment Research Council Isotope Geosciences Laboratory (Keyworth, Nottingham, England), where they were powdered. Oxygen (δ^18^O_carbonate_) and carbon (δ^13^C_carbonate_) isotope ratios in the carbonate fraction of enamel were determined according to the methodology outlined in Chenery et al. (2012, pp. 310): approximately 3 mg of clean powdered enamel was placed in glass vials which were sealed with septa and transferred to a hot block at 90**°**C on a Multiprep system (GV Instruments, Manchester, UK). Vials were evacuated and four drops of anhydrous phosphoric acid added and resultant CO_2_ collected cryogenically for 14 min. A GV IsoPrime dual inlet mass spectrometer was used to measure δ^18^O_carbonate_ and δ^13^C_carbonate_ values. Isotope ratios are reported as delta (δ) values, in parts per thousand (per mil; ‰) normalized to the VPDB scale using an in‐house carbonate reference material, Keyworth Carrera Marble (KCM) calibrated against NBS19 certified reference material. Analytical reproducibility for this run of KCM was ± 0.09 ‰ (1σ, *n* = 14) for δ^18^O and for δ^13^C ± 0.04 ‰ (1σ, *n* = 14)_._ δ^18^O_carbonate_ values were normalized to the VSMOW scale using the equation of Coplen, 1988 (VSMOW = 1.03091 x δ^18^O VPDB + 30.91) and the regression equation of Chenery et al. (2012: 310; δ^18^O_phosphate_ = 1.0322 x δ^18^O_carbonate_ – 9.6849) used to convert δ^18^O_carbonate_ values to δ^18^O_phosphate_. The error involved in calculating δ^18^O_phosphate_ is considered to be low (0.28 ‰, 1σ, Chenery et al., 2012, pp. 313).

Radiocarbon dating was conducted at the Scottish Universities Environmental Research Centre according to the methods described in Dunbar, Cook, Naysmith, Tripney, and Xu (2016) using collagen extracted from the root of one tooth of each dated individual (Tables 1 and 2). ^14^C results are reported as conventional radiocarbon ages, quoted in conventional years BP (before 1950 AD) (Millard, 2014; Stuiver & Polach 1977). Calibrated ages (Tables 1 and 2) were determined and Bayesian modelling undertaken using the University of Oxford Radiocarbon Accelerator Unit calibration program, OxCal v4.2 (Bronk Ramsey, 2009) and IntCal13 (Reimer et al., 2013). Calibrated date ranges are quoted in the form recommended by Millard (2014), rounded outwards by 10 years, and posterior density estimates are quoted in italics and rounded outwards to the nearest 5 years.

## 3 RESULTS

Eight of the nine human individuals from Ty Isaf are assigned by AMS radiocarbon dating to the mid 4^th^ to early 3^rd^ millennium BC (Table 1). The ninth is assigned to the Bronze Age. This individual, a child (39.190/324) aged approximately 3–4 years at death was excavated from the north side of the monument outside Chamber 2 and is dated to 1663–1506 cal BC (95% confidence, SUERC‐57789; Table 1). Enamel from the deciduous first and second molars of this individual gave ^87^Sr/^86^Sr values of 0.7141 and 0.7146, with strontium concentrations of 59 and 64 ppm respectively.

**Figure 4:**
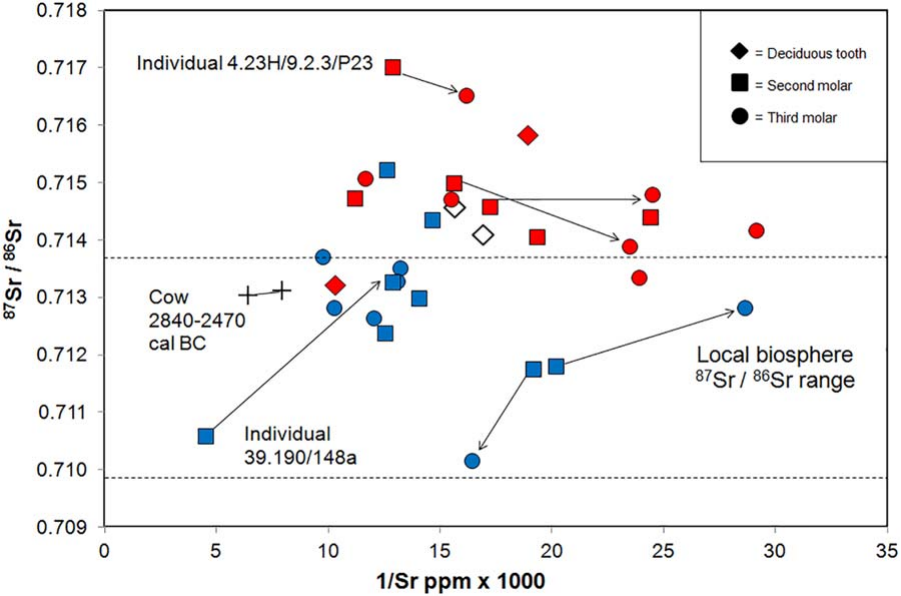
Plot of strontium isotope ratio versus the inverse of concentration (1/Sr ppm × 1000) for human and cattle enamel from Penywrylod (Talgarth) long cairn and Ty Isaf, Powys, Wales. Dashed lines delineate locally bioavailable ^87^Sr/^86^Sr values measured in plants growing within a radius of 10 miles (approximately 16 km) of both sites (Table 3). Arrows illustrate adjacent molars from the same individual. Red symbols: human individuals from Penywyrlod. Blue symbols: human individuals of mid to late 4^th^ millennium date sampled from Ty Isaf. Crosses: samples from the cusp and cervix of the third permanent premolar of a cow of later Neolithic date from Ty Isaf. Radiocarbon dates cited at 95% confidence. Open white symbols: first and second deciduous premolars of a child of Bronze Age date from Ty Isaf. Tooth types are denoted by the key in the upper right of the diagram. 2σ errors for ^87^Sr/^86^Sr are within the symbol

Tooth enamel from the human individuals of Neolithic date who were buried at Ty Isaf gave a range of ^87^Sr/^86^Sr values between 0.7101 and 0.7152 (mean 0.7128 ± 0.0013, 1σ, *n* = 15), with only two individuals recording strontium isotope ratios higher than 0.7140 (Figure 4): adult 39.190/310 excavated from Chamber 2 of the long cairn and sub‐adult 39.190/58 from Chamber 1, who have ^87^Sr/^86^Sr values of 0.7144 and 0.7152 respectively (Table 1). Fourteen of the fifteen teeth sampled from the group dated to the Neolithic at Ty Isaf have strontium concentrations between 35 and 102 ppm (mean 72 ± 18.0 ppm, 1σ). However, the second molar tooth of one individual (39.190/148a) in the group exhibits a particularly high strontium concentration of 222 ppm.

The loose third permanent premolar of the cow from Ty Isaf, excavated from the west compartment of Chamber 1, is dated to the late Neolithic (2831–2474 cal BC). Enamel sampled close to the cusp of the crown of this tooth gave an ^87^Sr/^86^Sr value of 0.7130 with a strontium concentration of 155 ppm (Table 1), while that taken close to the cervix had an ^87^Sr/^86^Sr value of 0.7131 and a strontium concentration of 126 ppm.

Individuals buried at Penywyrlod have strontium isotope ratios between 0.7132 and 0.7170 (mean 0.7148 ± 0.0011, 1σ, *n* = 15) and strontium concentrations between 34 and 97 ppm (mean 60.2 ± 19.6 ppm, 1σ, *n* = 15). In contrast to Ty Isaf, the majority of the teeth (12 of 15) sampled from Penywyrlod have strontium isotope ratios that are higher than 0.7140, including both individuals from excavated chambers NE II and III (see Figure 2) and the child whose remains were recovered from a cist in the lower part of the outer revetment wall close to the forecourt of the monument (Savory, 1984, pp. 22–23). Enamel samples from the second and third molars of one adult individual (74.23H/9.2.3/P23) from Penywyrlod gave particularly high ^87^Sr/^86^Sr values of 0.7170 and 0.7165 respectively (Figure 4). This individual was excavated from NE chamber II at Penywyrlod and is radiocarbon dated to 3770–3630 cal BC (95% confidence, SUERC‐63414; Table 2). In contrast to the ^87^Sr/^86^Sr values exhibited by this individual, the samples of modern plants that were collected within a radius of 10 miles (approximately 16 km) of Penywyrlod and Ty Isaf gave strontium isotope ratios between 0.7097 ‐ 0.7137 (mean 0.7107 ± 0.001, 1σ, *n* = 12, Table 3).

**Table 3:** ^87^Sr/^86^Sr values measured in modern plants growing on lithology of Lower Devonian age within 10 miles (approximately 16 km) of Penywyrlod and Ty Isaf

Latitude	Longitude	Sample number	Plant species	Superficial deposits	Bedrock geology	Formation	Geological period	^87^Sr/^86^Sr
51.94752	−3.42426	S‐WALES 36	Ivy (*Hedera helix*)	Glaciofluvial Sheet Deposits, Devensian	Argillaceous rocks and sandstone, interbedded	St Maughans Formation	Lower Devonian	0.710056
51.93542	−3.34336	S‐WALES 37	Ivy (*Hedera helix*) and Beech leaves (*Fagus sylvatica*)	Glaciofluvial Sheet Deposits, Devensian	Argillaceous rocks and sandstone, interbedded	St Maughans Formation	Lower Devonian	0.710418
51.92573	−3.34086	S‐WALES 38	Cow Parsley (*Anthriscus sylvestris*)	Glaciofluvial Fan Deposits, Devensian	Argillaceous rocks and sandstone, interbedded	St Maughans Formation	Lower Devonian	0.71137
51.90365	−3.29715	S‐WALES 39	Ivy (*Hedera helix*) and annuals	Till, Devensian	Sandstone and argillaceous rocks, interbedded	Senni Formation	Lower Devonian	0.710384
51.88732	−3.27605	S‐WALES 40	Hart's‐tongue fern (*Asplenium scolopendrium*)	None recorded	Sandstone and argillaceous rocks, interbedded	Senni Formation	Lower Devonian	0.710653
51.87312	−3.26747	S‐WALES 41	Hedgerow annuals	Glaciofluvial Deposits, Devensian	Sandstone and argillaceous rocks, interbedded	Senni Formation	Lower Devonian	0.711317
51.86806	−3.21373	S‐WALES 42	Ivy (*Hedera helix*)	Glaciofluvial Deposits, Devensian	Sandstone and argillaceous rocks, interbedded	Senni Formation	Lower Devonian	0.713664
51.93308	−3.12945	S‐WALES 43	Goose Grass (*Galium aparine*)	None recorded	Sandstone and argillaceous rocks, interbedded	Senni Formation	Lower Devonian	0.710166
51.87462	−3.10612	S‐WALES 44	Cow Parsley (*Anthriscus sylvestris*)	Till, Devensian	Sandstone and argillaceous rocks, interbedded	Senni Formation	Lower Devonian	0.709728
51.87568	−3.07297	S‐WALES 45	Lords‐and‐ladies (*Arum maculatum*)	Till, Devensian	Sandstone and argillaceous rocks, interbedded	Senni Formation	Lower Devonian	0.710393
51.88318	−3.03830	S‐WALES 46	Goose Grass (*Galium aparine*)	Glaciolacustrine Deposits, Devensian	Sandstone and argillaceous rocks, interbedded	Senni Formation	Lower Devonian	0.710621
51.88205	−2.98572	S‐WALES 47	Ivy (*Hedera helix*)	Glaciofluvial Deposits, Devensian	Argillaceous rocks and sandstone, interbedded	St Maughans Formation	Lower Devonian	0.709711

**Figure 5:**
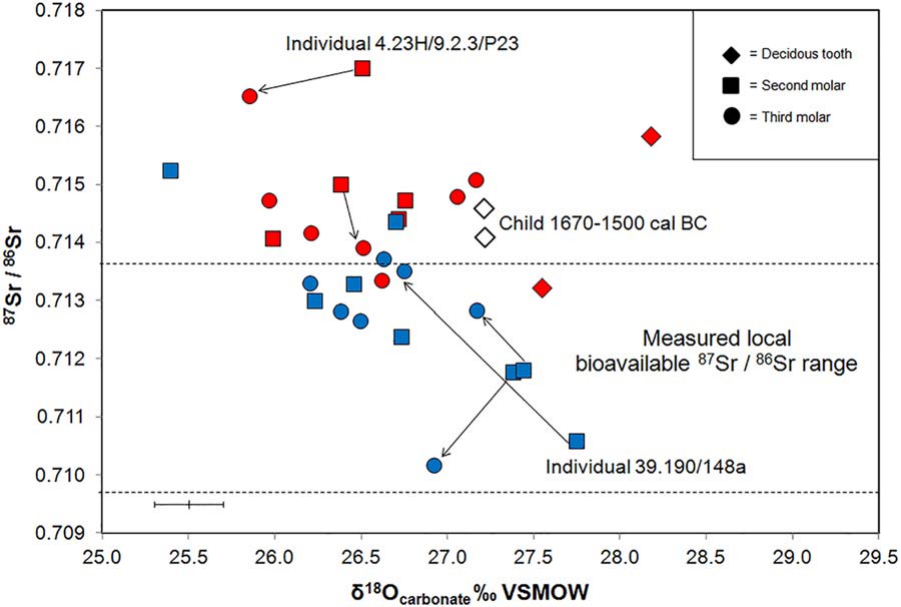
Plot of ^87^Sr/^86^Sr and δ^18^O_carbonate_ values of human and cattle enamel from Penywyrlod (Talgarth) and Ty Isaf, Powys, Wales. Dashed lines delineate locally bioavailable ^87^Sr/^86^Sr values measured in plants growing within a radius of 10 miles (approximately 16 km) of both sites (Table 3). Red symbols: human individuals from Penywyrlod long cairn. Blue symbols denote human individuals of mid to late 4^th^ millennium date from Ty Isaf. Open white symbols: first and second deciduous premolars of a child of Bronze Age date from Ty Isaf. Radiocarbon dates cited at 95% confidence. Tooth types are denoted by the key in the upper right of the diagram. Arrows illustrate adjacent molars from the same individual. 2σ errors for ^87^Sr/^86^Sr are within the symbol. 2σ errors for ^87^Sr/^86^Sr are within the symbol. Analytical error for δ^18^O_carbonate_ is shown as ± 0.2 ‰ (2σ)

δ^18^O_carbonate_ values of enamel from the second and third permanent molars of individuals of Neolithic date at Ty Isaf range between 25.4 and 27.7 ‰ (mean 26.7 ± 0.6 ‰, 1σ, *n* = 15; Figure 5): there are no discernible trends in enamel oxygen isotope composition according to excavation context or radiocarbon date within this group. Enamel samples from the first and second deciduous molars of the child of Bronze Age date (39.190/324) both gave δ^18^O_carbonate_ values of 27.2 ‰. δ^18^O_carbonate_ values of enamel from second and third permanent molars of individuals buried at Penywyrlod range between 25.9 and 27.2 ‰ (mean 26.5 ± 0.5 ‰, 1σ, *n* = 13), including adult individual (74.23H/9.2.3/P23) who has the highest strontium isotope ratio (0.7170) in the group and whose second and third molars gave δ^18^O_carbonate_ values of 26.5 ‰ and 25.9 ‰ respectively. Enamel from the second deciduous molars of two children from Penywyrlod (74.23H/9.18/P20 and 74.23H/9.7/P25) gave δ^18^O_carbonate_ values of 28.2 ‰ and 27.5 ‰ respectively.

Enamel δ^13^C_carbonate_ values of individuals sampled from Penywyrlod range between −18.0 and −15.0 ‰ (mean −16.0 ± 0.8 ‰, 1σ, *n* = 15). Enamel δ^13^C_carbonate_ values of individuals dated to the Neolithic at Ty Isaf range between −16.9 and −14.9 ‰ (mean −16.0 ± 0.6 ‰, 1σ, *n* = 15), while enamel from the first and second deciduous molars of the Bronze Age child from Ty Isaf gave δ^13^C_carbonate_ values of −15.2 ‰ and −15.6 ‰ respectively.

## 4 DISCUSSION

The majority of sampled individuals from Penywyrlod have ^87^Sr/^86^Sr values that are not consistent with the local biosphere range. They exhibit strontium isotope ratios above 0.7140 (Figure 4). These values exceed those measured in plants within a radius of 10 miles, approximately 16 km, of the site, which recorded ^87^Sr/^86^Sr values between 0.7097 ‐ 0.7137 (mean 0.7107 ± 0.001, 1σ, *n* = 12; Table 3). This suggests that individuals buried at Penywyrlod did not source their childhood diet locally. They may not, however, have obtained their diet from a great distance outside the local region: as discussed above, ^87^Sr/^86^Sr values up to 0.7162 have been recorded just over 35 miles (approximately 56 km) to the east of Penywyrlod in the Malvern Hills (Chenery et al., 2010, pp. 155). Although plants sampled in the Malverns routinely give lower ^87^Sr/^86^Sr values (mean 0.7128 ± 0.0040, 2σ, *n* = 13, Chenery et al., 2010, pp. 155–156) the possibility that these individuals obtained their childhood diet from this area cannot be excluded. Strontium isotope ratios between 0.7140 and 0.7150 have previously been recorded in human burials excavated close to the Malvern Hills, at the medieval cemeteries of Hereford Cathedral (Evans et al., 2012, pp. 756) and Blackfriars, Gloucester (Montgomery, 2002).

Alternatively, the possibility that individuals with ^87^Sr/^86^Sr values between approximately 0.7140 and 0.7150 spent their childhood further afield within central Wales could be considered. Currently available biosphere data from plants and water sampled on Ordovician and Silurian rocks in central Wales suggests that lithologies of this age in Wales are routinely associated with ^87^Sr/^86^Sr values lower than 0.7140 (see above, Evans et al., 2010 Montgomery et al., 2006, pp. 1628; Shand et al., 2007, pp. 254, 256). However, deep groundwaters sampled on lithology of Lower Palaeozoic age in central Wales have recorded ^87^Sr/^86^Sr values up to 0.7152 (Shand et al., 2007, pp. 256) and a single high value of 0.7147 has also been recorded in plants growing on Silurian mudstones in this region (FISH‐4, Evans et al., 2010). The possibility that individuals with strontium isotope ratios between approximately 0.7140 and 0.7150 sourced their diet from this area cannot be excluded.

Enamel from the second and third permanent molars of one individual in the burial population from Penywyrlod (adult 74.23H/9.2.3/P23; Figure 4) gave ^87^Sr/^86^Sr values of 0.7170 and 0.7165. These values are higher than all currently recorded biosphere values in England and Wales (Chenery et al., 2010, pp. 155; Evans et al., 2010). Current understanding of the methodology (as outlined in Sections 1 and 2) and all presently available measured biosphere ^87^Sr/^86^Sr data suggests that this individual obtained their childhood diet from outside England and Wales. The values this individual exhibits support the interpretation that this individual moved a significant distance after the formation of their third permanent molar tooth crown (i.e., after approximately 14.5 ± 0.5 years of age, AlQahtani et al., 2010; see Section 2.1).

This individual is dated to 3770–3630 cal BC (95% confidence, SUERC‐63414; Table 2), consistent with the radiocarbon result previously obtained from human bone excavated from Chamber NEII at Penywyrlod (3960–3640 cal BC, 95% confidence, OxCal v. 4.2; Britnell & Savory 1984, pp. 29). Bayesian modelling of currently available radiocarbon dates from southern Wales suggests that agriculture first began to develop in this region from the 38^th^ century BC, first appearing here between *3765 and 3655 cal BC*, 95% *probability*, probably in *3725–3675 cal BC, 68% probability* (Bayliss et al., 2011b: 548; OxCal v.3.10). The AMS radiocarbon evidence therefore suggests the individual could be contemporary in date with the first appearance of Neolithic material culture and practices in southern Wales. Recent comparative analysis of ceramics has been used to argue that Neolithic material culture and practices were introduced to western Britain by the arrival of groups from Lower Normandy and Brittany in north‐western France during the early 4^th^ millennium BC (Pioffet, 2015). The strontium isotope ratios that this individual exhibits could be consistent with such an origin: plants in Lower Normandy, Brittany and Pays de la Loire give ^87^Sr/^86^Sr values higher than 0.7165 (Négrel & Pauwels, 2004; Willmes et al., 2013). Both the strontium isotope results and radiocarbon dating may therefore support the hypothesis that development of agriculture in southern Wales was associated with arrival of migrant individuals from outside the region during the early 4^th^ millennium BC. The oxygen isotope ratios that the individual exhibits could also be consistent with an origin in north‐western France (see below).

Biosphere ^87^Sr/^86^Sr values higher than 0.7165, such as those this individual exhibits, have been recorded in regions further afield, in areas such as the Cairngorm mountains in north‐east Scotland, over 500 km from Penywyrlod (Evans et al., 2010). However, current Bayesian modelling suggests that Neolithic material culture and practices may only recently have become established within this region of Scotland at this time: it is argued that they appeared here in the decades around 3800 cal BC (Bayliss et al., 2011a, pp. 822–824, 838–840). ^87^Sr/^86^Sr values close to 0.7170 have similarly been recorded in Northern Ireland (Snoeck et al., 2016). However, with the exception of Ferriter's Cove in the far south‐west of Ireland, where cattle bone in Mesolithic contexts has been interpreted as a failed episode of colonization during the mid 5^th^ millennium BC (Sheridan, 2010a; Woodman, Andersen, & Finlay, 1999) and Magheraboy causewayed enclosure, Co. Sligo, the dating of which is currently regarded as problematic, there is at present limited evidence to suggest the Neolithic was established in Ireland any earlier than the 38^th^ century BC (Bayliss et al., 2011a, pp. 805–808; Cooney et al., 2011, pp. 663–668; Schulting, Murphy, Jones, & Warren, 2012, pp. 31; Whitehouse et al., 2014).

Although areas in closer geographic proximity within England and Wales have been considered (see above) to explain the prevalence of ^87^Sr/^86^Sr values between 0.7140 and 0.7150 within the population at Penywyrlod, the possibility that the majority of individuals who are buried at this site spent their childhood further afield must also be considered. Plants sampled in regions such as north‐western France can, for example, record a range of values comparable to those exhibited by this burial group (i.e., between approximately 0.7140 and 0.7170). As such, it is possible that the majority of those buried at Penywyrlod could have obtained their childhood diet outside England and Wales: the possibility that they originated from a similar region to the individual who exhibits the highest values in the group cannot be excluded.

In contrast to Penywyrlod, all individuals in the group sampled from Ty Isaf have calibrated radiocarbon dates that fall after 3650 cal BC (Table 1) and therefore post‐date the first appearance of Neolithic material culture and practices in southern Wales (see above; Bayliss et al., 2011a, pp. 738, 2011b, pp. 548). The majority have strontium isotope ratios that are consistent with local biosphere values and could therefore support the interpretation that these individuals obtained their childhood diet locally. Irrespective of calibrated date or excavation context, the strontium isotope ratios exhibited by most of this group fall within the range of values measured within 10 miles (approximately 16 km) of the site (between 0.7097 and 0.7137, mean 0.7107 ± 0.001, 1σ, *n* = 12, Table 3; Figure 4), although the possibility that individuals obtained their childhood diet further afield, from areas with a similar biosphere range, cannot be excluded. For example, sandstones of Devonian age in east Somerset and north Devon also record ^87^Sr/^86^Sr values between approximately 0.7120 and 0.7130 (Evans et al., 2010; Montgomery et al., 2006, pp. 1628). Individuals who exhibit these values could equally have obtained their childhood diet from these regions. Likewise, although individuals with strontium isotope ratios between approximately 0.7100 and 0.7110 plot within range of biosphere ^87^Sr/^86^Sr values measured locally, other areas, either within Wales (e.g., coastal regions of Pembrokeshire), or further afield (e.g., Cornwall, England), also record similar biosphere values (ibid.). With the exception of individual 39.190/59 who has a ^87^Sr/^86^Sr value of 0.7102, strontium isotope results from Ty Isaf (and Penywyrlod) appear to rule out the possibility that individuals obtained a significant component of their childhood diet from south‐central and eastern regions of England. In the latter regions, lithologies of Jurassic and Cretaceous age routinely record biosphere values below ^87^Sr/^86^Sr 0.7105 (Warham, 2011, pp. 79; see Figure 1). Only two individuals in the burial group from Ty Isaf have strontium isotope ratios higher than 0.7140. Both adult 39.190/310 and sub‐adult 39.190/58 were exposed to more radiogenic sources of strontium than are known to be bioavailable locally during formation of their second permanent molar crown, between approximately 2.5 ± 0.5 years to 8.5 ± 0.5 years of age (AlQahtani et al., 2010; Hillson, 2014, pp. 31, 55–56). These two individuals could have derived their childhood diet from similar source areas to those discussed above for the group sampled from Penywyrlod.

Penywyrlod and Ty Isaf are part of a cluster of early Neolithic long cairns in the Black Mountains. While their construction appears to reflect a common theme, it is argued that differences in the morphology of these monuments may indicate they were built and used by different social groups (Wysocki & Whittle, 2000, pp. 600). The strontium isotope results could support this suggestion. While they are situated less than 5 miles (8 km) apart, these monuments differ in their morphology and the ^87^Sr/^86^Sr results are consistent with the two burial groups having obtained their diet from different geographical locations. The majority of those buried at Penywyrlod appear to have obtained their childhood diet from outside the immediate area in which they were buried, whereas results from Ty Isaf could support the hypothesis that individuals sourced their diet locally. It is possible that this contrast may be the result of a difference in the chronology of the two burial groups as, unlike the individual from Penywyrlod who was radiocarbon dated by this study, all individuals from Ty Isaf post‐date 3650 cal BC. Further radiocarbon dates are being sought from Penywyrlod to examine this possibility.

The rules defining who was selected for burial in such monuments have also often been debated (e.g., Whittle et al., 2007, pp. 134). The results of this study suggest that, rather than acting as a focus for burial of individuals who sourced their diet from a diverse range of geographical areas, the individuals who were chosen for burial within each monument could have obtained their diet from a similar geographical location.

Where adjacent consecutively mineralizing second and third molar teeth were available for sampling from Ty Isaf, the majority of tooth pairs (6 of 7) have values that plot within the local biosphere range (the exception being individual 39.190/310, discussed above, who may have obtained their diet outside the local area during childhood). For example, adult 39–190/312 has adjacent second and third molar teeth with ^87^Sr/^86^Sr values of 0.7130 and 0.7126 respectively. This could be consistent with the individual having obtained their diet from a similar geographical location, such as the local area, for a prolonged period during early life. Alternatively, however, they could have sourced their diet from different geographical locations that conferred a similar averaged ^87^Sr/^86^Sr value (close to 0.7130) during the formation of each tooth. Similarly, individuals buried at Penywyrlod who have high strontium isotope ratios (above 0.7140) on each of their adjacent teeth (illustrated with arrows in Figure 4) could either have obtained their diet from one of the areas discussed as potential sources for this value over a prolonged period during early life; or alternatively they could have sourced their diet from several different geographical locations that conferred a similar averaged ^87^Sr/^86^Sr value (i.e., higher than approximately 0.7140).

**Figure 6:**
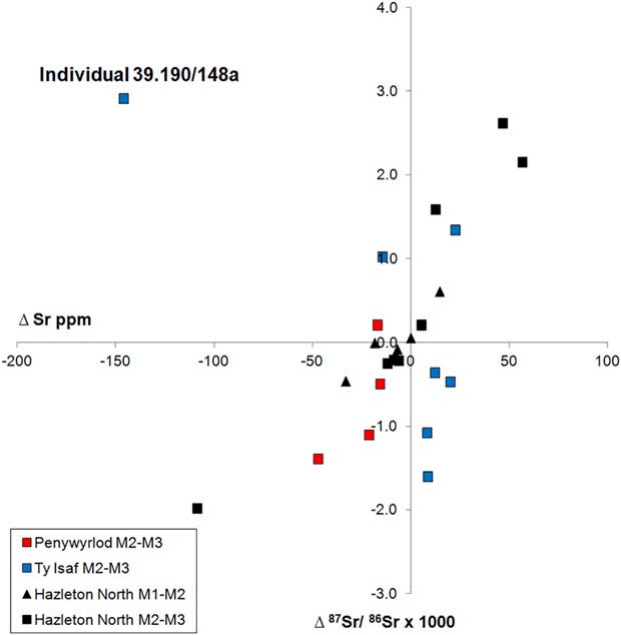
Plot of the difference in strontium isotope ratio (Δ ^87^Sr/^86^Sr x 1000) and elemental concentration (Δ Sr ppm) between adjacent permanent molar teeth of individuals excavated from Penywyrlod (Talgarth) individuals of Neolithic date from Ty Isaf long cairn, Powys, Wales. Red symbols: Penywyrlod; Blue symbols: Ty Isaf. Black symbols: Hazleton North long cairn, Gloucestershire, England. Triangles denote the difference in strontium isotope ratio and Sr ppm between first and second molars. Squares denote the difference in strontium isotope ratio and elemental concentration between second and third molars. 2σ errors for ^87^Sr/^86^Sr are within the symbol

**Figure 7:**
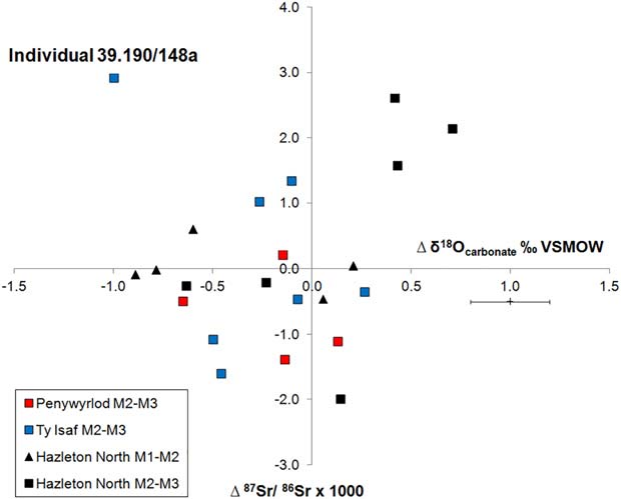
Plot of the difference in oxygen (Δ δ^18^O_carbonate_ ‰ VSMOW) and strontium (Δ ^87^Sr/^86^Sr x 1000) isotope ratios between adjacent permanent molar teeth of individuals excavated from Penywyrlod (Talgarth) and individuals of Neolithic date from Ty Isaf long cairn, Powys, Wales. Red symbols: Penywyrlod; Blue symbols: Ty Isaf. Black symbols: Hazleton North long cairn, Gloucestershire, England. Triangles denote the difference in strontium and oxygen isotope ratio between first and second molars. Squares denote the difference in strontium and oxygen isotope ratio between second and third molars. 2σ errors for ^87^Sr/^86^Sr are within the symbol. Analytical error for δ^18^O_carbonate_ is shown as ± 0.2 ‰ (2σ)

Individual 39.190/148a (labelled on Figures 4 and 6) from Ty Isaf exhibits the largest shift in isotope ratio of all sampled individuals in the present study: from an ^87^Sr/^86^Sr value of 0.7106 in their second permanent molar enamel to a value of 0.7135 in enamel from their third permanent molar. Although this shift is accompanied by a change in oxygen isotope ratio (Figure 7) and a drop in strontium concentration, from 222 to 76 ppm (discussed below), since both the ^87^Sr/^86^Sr values fall within the measured local biosphere range, the possibility that this individual sourced their diet from the local area throughout the period both teeth were forming cannot be excluded. In contrast, a change in the geographical location from which individuals obtained their diet can be inferred from shifts in strontium isotope ratio and elemental concentration at Hazleton North long cairn, Gloucestershire (Figures 6 and 7). At this site, several individuals exhibited a change in strontium isotope ratios from a value lower than 0.7085 to higher than 0.7105 between adjacent teeth. In southern Britain, with the possible exception of the Lizard Peninsula in Cornwall, lithologies that routinely give measured biosphere ^87^Sr/^86^Sr values below 0.7085 are geographically separated from those that give values higher than 0.7105 and the shift between these two values can therefore be interpreted to indicate that individuals changed the location from which they obtained their diet during childhood (Evans et al., 2010; Neil, Evans, Montgomery, & Scarre, 2016a).

If δ^18^O_carbonate_ results are converted to δ^18^O_phosphate_ values using the equation of Chenery et al. (2012), individuals of Neolithic date from Ty Isaf with strontium isotope ratios comparable to the local measured biosphere range have δ^18^O_phosphate_ values between 17.4 and 19.0 ‰ (mean 18.0 ± 0.5 ‰, 1σ, *n* = 13). This comparison could be used to suggest that the majority of the group from Ty Isaf obtained their diet from the local region in which they were buried, as their δ^18^O_phosphate_ values fall close to the mean value that Evans et al. (2012, pp. 759; mean 18.2 ‰ ± 1.0 ‰, 2σ) argue to be representative of occupation of western Britain where the site is located. At Hazleton North long cairn, also located in western Britain, conversion of δ^18^O_carbonate_ values of second and third permanent molars gave a similar range of δ^18^O_phosphate_ values (17.6 ‐ 18.9 ‰, mean 18.2 ± 0.4 ‰, *n* = 20, 1σ; Neil et al., 2016a). In both cases, however, this comparison assumes that δ^18^O values in human enamel directly reflect geographical variation in the oxygen isotope composition of drinking waters and that values have not been significantly elevated as a result of culinary practice (e.g., Brettell et al., 2012b), or by consumption of fluids that have undergone fractionation through biological processes (e.g., cow's milk; Camin et al., 2008, pp. 1695; Kornexl et al., 1997, pp. 22; Lin, Rau, Chen, Chou, & Fu, 2003, pp. 2193).

Only one individual in the sampled group from Ty Isaf has an δ^18^O_phosphate_ value that falls outside the range suggested to represent occupation of western Britain. This individual, (39.190/58; Table 1), is one of the two buried at Ty Isaf who has a strontium isotope ratio (0.7152) higher than the local biosphere range. Their δ^18^O_phosphate_ value of 16.5 ‰ is lower than the range of values considered to represent occupation of western Britain (17.2 to 19.2 ‰; mean 18.2 ‰ ± 1.0 ‰, 2σ, Evans et al., 2012, pp. 759). However, it does fall within the range for eastern Britain (15.9 to 18.5 ‰; mean 17.2 ± 1.3 ‰, 2σ; ibid.). The combination of oxygen isotope and strontium isotope ratios exhibited by this particular individual, who is dated to 3520–3340 cal BC (Table 1), could therefore suggest that they derived their childhood diet from a region further afield, such as north‐east Scotland which records biosphere ^87^Sr/^86^Sr values higher than 0.7140 and low δ^18^O values in ground waters (Darling et al., 2003, pp. 189, 191; Evans et al., 2010). However, before reaching this conclusion the possibility that there is greater spatial and temporal variability in the oxygen isotope composition of groundwaters of closer regions that can record biosphere ^87^Sr/^86^Sr values higher than 0.7140 (e.g., central Wales) might be considered. It is possible that altitude effects, for example, may induce greater local variability in the isotopic composition of groundwaters within Wales (Darling et al., 2003, pp. 189), resulting in lower δ^18^O values in areas of higher elevation (Mook, 2005, pp. 94). In addition, although it has been argued that climatic variation during the Holocene is unlikely to have been sufficiently significant to have influenced δ^18^O values exhibited by human populations (Evans et al., 2012, pp. 758), the precise effect of local climatic variability on δ^18^O values in groundwaters over time (e.g., at a seasonal, inter‐annual or decadal scale) in north‐western Europe remains poorly understood and new proxies are currently being sought to study localized variation in past precipitation levels and temperature (e.g., Young et al., 2015, 2012).

δ^18^O_phosphate_ values for second and third molar teeth of individuals excavated from Penywyrlod range between 17.0 and 18.4 ‰ (mean 17.7 ± 0.5 ‰, 1σ, *n* = 13). These values are therefore comparable to the ranges proposed by Evans et al. (2012) to represent occupation of Britain. However, as discussed in the introduction, oxygen isotope ranges of burial populations excavated in Britain overlap with those of burial groups found on the European mainland (see Lightfoot & O'Connell 2016). Modern groundwaters and precipitation in north‐western France can record a comparable range of δ^18^O values to those in Wales (between approximately −5 to −7 ‰, Darling et al., 2003; IAEA/WMO, 2016; Lécolle, 1985; Millot, Petelet‐Giraud, Guerrot, & Négrel, 2010) and archaeological populations excavated in Lower Normandy (e.g., Brettell et al., 2012a, pp. 127 & 132) have also recorded values comparable to those exhibited by individuals buried at Penywyrlod.

The deciduous molars of two children sampled by this study (from Penywyrlod) have not been included in the above comparison of oxygen isotope ranges, as they begin formation *in utero* (AlQahtani et al., 2010) and, following birth, values within these teeth may be influenced by consumption of breast milk, which has a higher δ^18^O value relative to fluids consumed by the mother (Britton, Fuller, Tütken, Mays, & Richards, 2015; Roberts et al., 1988; Wright & Schwarcz, 1998). The deciduous second molar of child 74.23H/9.18/P20 from Penywyrlod (Figure 5) exhibits the highest δ^18^O_carbonate_ value (28.2 ‰; δ^18^O_phosphate_ 19.4 ‰) of all the individuals sampled in the present study: the possibility that this is a consequence of breastfeeding cannot be excluded.

Enamel δ^13^C_carbonate_ values of individuals from Penywyrlod range between −18.0 and −15.0 ‰ (mean −16.0 ± 0.8 ‰, *n* = 15), while enamel δ^13^C_carbonate_ values of individuals dated to the Neolithic at Ty Isaf range between −16.9 and −14.9 ‰ (mean −16.0 ± 0.6 ‰, *n* = 15). These values fall within the range that may be predicted for a diet dominated by C_3_ terrestrial resources (between approximately −17.0 and −14.0 ‰; Froehle et al., 2012; Kellner & Schoeninger, 2007). The majority of human individuals sampled at both Penywyrlod and Ty Isaf have strontium concentrations comparable to those exhibited by the early Neolithic burial population at Hazleton North, where concentrations ranged between 22 and 144 ppm (mean 54 ± 25 ppm, *n* = 35, Neil et al., 2016a). Strontium concentrations recorded at Penywyrlod and Ty Isaf are also close to the median value of 84 ppm (*n* = 614) reported by Evans et al. (2012, pp. 756) for archaeological populations dating from the Neolithic to the 19^th^ century in Britain as a whole. However, one individual (39.190/148a) at Ty Isaf exhibited a particularly high strontium concentration: enamel from their second permanent molar tooth had a value of 222 ppm (Figures 4 and 6). Enamel from their third molar, however, recorded a strontium concentration of 76 ppm, comparable to the rest of the group.

Multiple factors may be influential in determining strontium concentrations in mammalian tissues (see reviews by Burton & Wright, 1995, pp. 280; Montgomery 2002, pp. 36–39, 2010, pp. 328), including the trophic level of an individual. Progressive biopurification, discrimination against strontium in favour of calcium (in the digestive tract, Sips, Barto, Netelenbos, & Van der Vijgh, 1997, and kidneys, Kobayashi & Suzuki, 1990) occurs at successive trophic levels within a food chain (Blum et al., 2000; Burton, Price, & Middleton, 1999). As a consequence, herbivores can record higher strontium concentrations than human populations. Strontium concentrations in cattle enamel of prehistoric date excavated in Britain frequently exceed 150 ppm (e.g., Minniti, Valenzuela‐Lamas, Evans, & Albarella, 2014, pp. 310; Towers, Montgomery, Evans, Jay, & Parker Pearson, 2010, pp. 511; Viner et al., 2010; also see Neil et al. 2016). Enamel samples from the later Neolithic cow tooth at Ty Isaf (dated to 2840–2470 cal BC; see below; Table 1) gave strontium concentrations of 126 and 155 ppm, consistent with those reported for later Neolithic cattle of similar date sampled at Durrington Walls, Wiltshire (Viner et al., 2010, pp. 2185).

One factor that has been invoked to explain the particularly high strontium concentrations observed within human archaeological populations in an Atlantic facing island such as Britain is the potential for marine‐derived strontium to contribute to diet, owing to the high concentration of strontium in seawater (Odum, 1957) and aerial deposition through seaspray (e.g., Whipkey, Capo, Chadwick, & Stewart, 2000). High strontium concentrations are also recorded in association with other types of environment (e.g., hot arid climates, e.g., Buzon, Simonetti, & Creaser, 2007), but in Britain, high strontium concentrations are frequently recorded in populations excavated at coastal island locations (e.g., Armit, Shapland, Montgomery, & Beaumont, 2015, pp. 8–9; also see Hemer, Evans, Chenery, & Lamb, 2014, pp. 245; Montgomery, Evans, & Cooper, 2007, pp. 1509). It has been argued that high strontium concentrations may either result from individuals obtaining dietary resources from geographical areas that receive high levels of seaspray, or from exploitation of seaweed as fertilizer and deliberate strategies that lead to increased salt intake (e.g., preserving food with sea salt, Montgomery, Evans, & Neighbour, 2003, pp. 651; Montgomery, 2010, pp. 334). The shift in strontium concentration from 222 to 76 ppm that the individual from Ty Isaf exhibits is accompanied by both a shift in strontium isotope ratio from 0.7106 to 0.7135 and a shift in oxygen isotope ratio (see Figures 4, 5, 6, 7). This could tentatively provide support for the argument that the individual changed the location from which they obtained their diet during childhood (although see discussion of the caveats relating to the local ^87^Sr/^86^Sr biosphere range, above).

Using Bayesian modelling of seven radiocarbon determinations obtained from human bone within the rotunda, Bayliss et al. (2011b, pp. 537, 546–547) suggest that use of Ty Isaf for burial began in *3825–3345* cal BC (*95% probability*), probably in *3520–3360* cal BC (*68% probability*) and that burial activity ended between *3365–2780* cal BC (*95% probability*), probably in *3355–3065* (*68% probability*; ibid. re‐calibrated using OxCal v4.2, IntCal13). However, it should be noted that contra Bayliss et al. (2011b, pp. 546–547), as these radiocarbon determinations were obtained from disarticulated bones, they date the deaths of the individuals who were sampled, rather than providing a date for burial activity or use of the monument itself. Like the individuals dated by the present study (Table 1) all those dated by Bayliss et al. from the rotunda have calibrated radiocarbon dates that fall after 3650 cal BC (ibid., 2011b, pp. 537). Bayesian modelling of the sample of early Neolithic long cairns and barrows in southern Britain for which radiocarbon chronologies have currently been constructed suggests the majority were only used for relatively short periods of time, often for only two to three generations, during the 4^th^ millennium BC, between approximately 3750–3550 cal BC (Whittle et al., 2007, pp. 129, 131, and 137). When modelling dates from the rotunda, Bayliss et al. (2011b) therefore excluded two individuals who were dated to the earlier 3^rd^ millennium BC. These individuals were assumed to belong to a distinct phase of secondary burial activity during the later Neolithic (ibid. 546), rather than being indicative of long‐lived and episodic use of the monument over an extended time period, spanning the mid 4^th^ to earlier 3^rd^ millennium BC. The latter scenario cannot, however, be ruled out. Documentation of the precise stratigraphic context of remains excavated from Ty Isaf is limited. However, that which is available does not support the argument that these individuals belong to a discrete phase of later Neolithic secondary burial activity as remains dated to the 3^rd^ millennium BC were found in both the lower and upper stratigraphic levels in the rotunda (OxA‐14248 dated to 2900–2670 cal BC and OxA‐14250 dated to 2860–2490 cal BC respectively, 95% confidence, Bayliss et al., 2011b, pp. 537; calibrated using OxCal v4.2, IntCal13).

**Figure 8:**
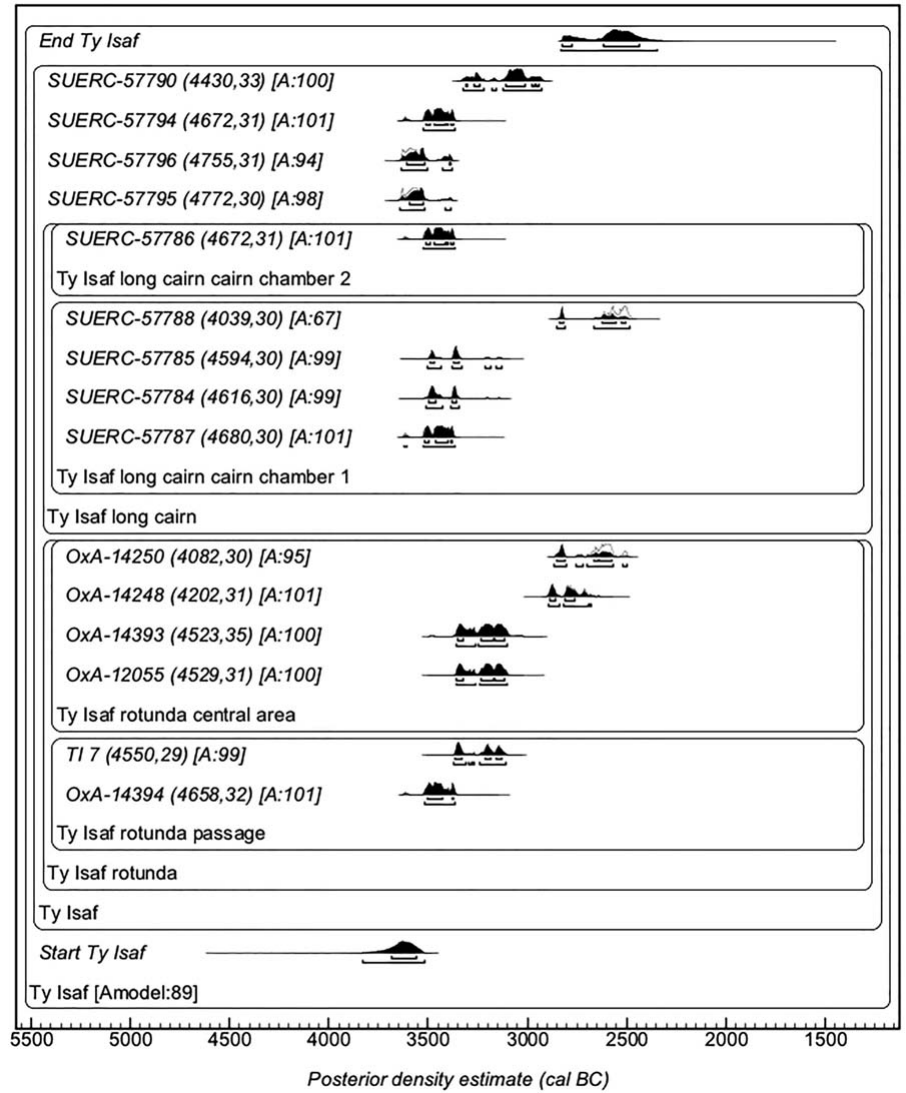
Probability distributions of radiocarbon dates from Ty Isaf. Determined using OxCal v4.2 (Bronk Ramsey, 2009) and IntCal13 (Reimer et al., 2013). Brackets within the diagram and OxCal keywords define the overall model. Radiocarbon dates obtained on human bone from the rotunda derive from Bayliss et al. (2011b, pp. 537). Individual 39.190/324 who is dated to the Bronze Age (1670–1510 cal BC; Table 1) has been excluded from this model

Calibrated date ranges of human remains selected for sampling by the present study also span the mid 4^th^ to earlier 3^rd^ millennium BC, with the latest human individual dating to 3330–2920 cal BC (95% confidence; 39.190/59; SUERC‐57790; Table 1). This individual is of undocumented context and could have been excavated from either the rotunda or the long cairn (see Section 2.1). Bayesian modelling of the radiocarbon determinations obtained from four human individuals excavated from the lateral chambers of the long cairn (Table 1) suggests that they died between *3630 and 3365 (95% probability*, or *3545–3460*, *68% probability)* and *3500–3235* cal BC *(95% probability*, or *3485–3335* cal BC, *68% probability)*. However, this model excludes the radiocarbon determination obtained from the cattle tooth, found in Chamber 1 of the long cairn, which is dated to the 3^rd^ millennium BC (2840–2470 cal BC, 95% confidence; SUERC‐57788; Table 1). Modelling of all currently available radiocarbon determinations of Neolithic age obtained from Ty Isaf (Figure 8; excluding child 39.190/324 dated to the Bronze Age) suggests that individuals who were buried at the site died between 3835 and 3515 cal BC (*95% probability*, or *3690–3555 cal BC*, *68% probability*) and 2830–2345 cal BC (*95% probability*, or *2825–2435 cal BC*, *68% probability*) and the possibility that the monument continued to be used for burial throughout this period cannot be ruled out.

Like the majority of the Neolithic human population buried at Ty Isaf, enamel from the cattle tooth exhibits strontium isotope ratios that are comparable to the local biosphere range (Figure 4) and could be interpreted to indicate that the animal was raised locally. The animal derived strontium from sources that conferred a similar averaged value during both earlier and later stages of tooth formation, with samples from the cusp and cervix both giving ^87^Sr/^86^Sr values close to 0.7130. In contrast to this animal and the majority of the Neolithic human population buried at Ty Isaf, tooth enamel from the child of Bronze Age date (Figures 4 and 5) gave ^87^Sr/^86^Sr values of 0.7141 and 0.7146 that are higher than the current recorded local biosphere range. This suggests that this individual was exposed to non‐local sources of strontium and obtained their diet from further afield, with the closest areas to record comparable values being the Malvern Hills and central Wales, as discussed above. These isotope ratios were recorded in enamel of deciduous first and second molars which begin formation *in utero*. As such, it is possible that they could reflect dietary resources that were exploited by the mother (Montgomery, 2002, pp. 44). If δ^18^O_carbonate_ results from this individual are converted to δ^18^O_phosphate_ values using the equation of Chenery et al. (2012), both teeth give values of 18.4 ‰. This falls close to the mean value that Evans et al. (2012, pp. 759) argue to represent occupation of western Britain. However, as formation of deciduous molars continues in the months following birth, it is possible that oxygen isotope ratios in enamel from these teeth could also be influenced by consumption of breast milk, which may confer higher δ^18^O values than drinking water (Britton et al., 2015; Roberts et al., 1988; Wright & Schwarcz, 1998). The remains of this individual, dated to 1670–1500 cal BC (95% confidence; SUERC‐57789), were found outside Chamber 2 of the long cairn (Table 1). As prominent features in the landscape, Neolithic long cairns were sometimes re‐used for burial in later periods (Darvill 2004, pp. 214–232). The AMS radiocarbon results support the suggestion of the excavator that Ty Isaf was re‐used for burial during the Bronze Age (Grimes, 1939, pp. 135–136).

## 5 CONCLUSIONS

The majority of individuals buried at Penywyrlod have strontium isotope ratios that exceed the local biosphere range, suggesting they sourced their childhood diet outside the local region. Biosphere ^87^Sr/^86^Sr values comparable to those exhibited by this group (i.e., between 0.7140 and 0.7150) have been recorded in the Malvern Hills in England and central Wales. However, the possibility that the majority of these individuals obtained their childhood diet in areas further afield cannot be excluded.

One individual in the burial group from Penywyrlod (74.23H/9.2.3/P23) has strontium isotope ratios (0.7165 and 0.7170) that exceed all currently recorded biosphere values in England and Wales. All current understanding of the methodology suggests this individual cannot have obtained their childhood diet from England and Wales. The period to which this individual is dated, to between 3770 and 3630 cal BC (95% confidence), is consistent with recent dating estimates for the first appearance of agriculture in southern Wales between *3765–3655 cal BC* (*95% probability*; *3725–3675 cal BC, 68% probability*, Bayliss et al., 2011b, pp. 548). The individual is associated with Neolithic material culture: monuments of the type in which this individual was buried derive from Continental traditions (Scarre, 2015). The ^87^Sr/^86^Sr values they exhibit are comparable to ranges bioavailable in Lower Normandy, Brittany and Pays de la Loire in north‐western France (Négrel & Pauwels, 2004; Willmes et al., 2013) and the results may plausibly be interpreted to suggest that this individual obtained their childhood diet within this region. The results could support the argument that development of agriculture in southern Wales was associated with the arrival of migrant individuals from outside the region.

Biosphere ^87^Sr/^86^Sr values higher than 0.7165, such as those this individual exhibits, have been recorded in regions further afield, in areas such as the Cairngorm mountains in north‐east Scotland, over 500 km from Penywyrlod (Evans et al., 2010). However, current Bayesian modelling suggests that Neolithic material culture and practices may only recently have become established within this region of Scotland at this time, beginning to appear in this area from the decades around 3800 cal BC (Bayliss et al., 2011a). Biosphere ^87^Sr/^86^Sr values close to 0.7170 have similarly been recorded in Northern Ireland (Snoeck et al., 2016). However, the attribution of this individual to this area may also be problematic on archaeological grounds as at present there is limited evidence to suggest that Neolithic material culture and practices were established in Ireland any earlier than the 38^th^ century BC (Bayliss et al., 2011a, pp. 805–808; Cooney et al., 2011, pp. 663–668; Schulting et al., 2012, pp. 31; Whitehouse et al., 2014).

Unlike the burial group at Penywyrlod, the majority of individuals who were buried at Ty Isaf exhibit strontium isotope ratios that are consistent with the local biosphere range and could have sourced their diet locally. In contrast to individual 74.23H/9.2.3/P23 from Penywyrlod, none of the individuals sampled at Ty Isaf have calibrated radiocarbon dates that fall within the period in which agriculture is considered to have been first initiated in southern Wales. The program of radiocarbon dating undertaken by the present study at Ty Isaf also identified the presence of cattle remains dated to the later Neolithic and a child of Bronze Age date, suggesting that this monument may have remained of importance to communities beyond the 4^th^ millennium BC.

## ACKNOWLEDGMENTS

We would like to thank Hilary Sloane (NERC Isotope Geosciences Laboratory) for analytical support; Lucie Johnson for discussion of British biosphere data; Christophe Snoeck for access to data from Ireland in advance of publication; Adam Gwilt, Dr Steve Burrow, Mary Davies, Jodie Deacon and Evan Chapman at the National Museum Wales for permission to sample collections and Bill Britnell (Clwyd‐Powys Archaeological Trust) for providing Figures 2 and 3.

## AUTHOR CONTRIBUTION

S.N. designed the research; S.N., J.E. and G.C. undertook the analysis; J.E. provided strontium isotope results from locally collected modern plants (Table 3); S.N. wrote the paper. All authors discussed drafts of the paper and approved the final manuscript. S.N. was funded through a Durham University Doctoral Studentship. Strontium and oxygen isotope analysis was funded through NIGFSC grant IP‐1290‐0512 awarded to J.M. Radiocarbon dating was funded by a Prehistoric Society radiocarbon dating award (to S.N.), a Durham University Projects and Initiatives Grants Scheme award (to S.N.) and the Scottish Universities Environmental Research Centre.
